# Reconstitution of RNA Polymerase I Upstream Activating Factor and the Roles of Histones H3 and H4 in Complex Assembly

**DOI:** 10.1016/j.jmb.2018.01.003

**Published:** 2018-01-31

**Authors:** Marissa L. Smith, Weidong Cui, Ashleigh J. Jackobel, Nancy Walker-Kopp, Bruce A. Knutson

**Affiliations:** 1 - Department of Biochemistry and Molecular Biology, SUNY Upstate Medical University, 750 East Adams Street, Syracuse, NY 13210, United States; 2 - Department of Chemistry, Washington University in St. Louis, One Brookings Drive, St. Louis, MO 63130, United States

**Keywords:** UAF, RNA polymerase I, histone H3, histone H4, complex integrity

## Abstract

RNA polymerase I (Pol I) transcription in *Saccharomyces cerevisiae* requires four separate factors that recruit Pol I to the promoter to form a pre-initiation complex. Upstream Activating Factor (UAF) is one of two multi-subunit complexes that regulate pre-initiation complex formation by binding to the ribosomal DNA promoter and by stimulating recruitment of downstream Pol I factors. UAF is composed of Rrn9, Rrn5, Rrn10, Uaf30, and histones H3 and H4. We developed a recombinant *Escherichia coli*-based system to coexpress and purify transcriptionally active UAF complex and to investigate the importance of each subunit in complex formation. We found that no single subunit is required for UAF assembly, including histones H3 and H4. We also demonstrate that histone H3 is able to interact with each UAF-specific subunit, and show that there are at least two copies of histone H3 and one copy of H4 present in the complex. Together, our results provide a new model suggesting that UAF contains a hybrid H3–H4 tetramer-like subcomplex.

## Introduction

In *Saccharomyces cerevisiae*, RNA polymerase I (Pol I) transcription of the 35S pre-ribosomal RNA (rRNA) is the first step of ribosome biogenesis and must be tightly regulated to maintain proper ribosome levels in the cell. Pol I transcription initiation is coordinated by four Pol I initiation factors that include the Upstream Activating Factor (UAF), TATA-binding protein (TBP) [1–6], Core Factor (*CF*) [1,7–9], and Rrn3 [10,11]. These factors work together to recruit Pol I to the ribosomal DNA (rDNA) promoter and to initiate transcription.

UAF is a six-subunit complex composed of Rrn9, Rrn5, Uaf30, Rrn10, and histones H3 and H4 [12–14]. UAF targets the upstream activating sequence (UAS) at the rDNA promoter which lies from approximately −155 to −60 relative to the transcription start site [12,15–17]. Although UAF is not required for Pol I transcription *in vitro*, UAF is required for *in vivo* Pol I activity [1,18–20]. Deletion of *rrn5*, *rrn9*, or *rrn10* renders UAF non-functional, resulting in a loss of Pol I transcription and significant slow growth defects [12,19]. UAF also provides a barrier to Pol II transcription upstream of the rDNA promoter. In the absence of functional UAF, cells gain the ability to transcribe the rDNA using Pol II instead of Pol I, which is known as polymerase switching [19,20].

The presence of histones H3 and H4 in the UAF complex is particularly interesting as they are two of the four core histone proteins that make up the eukaryotic nucleosome. Two copies of histones H3 and H4 bind to form a stable tetramer, which associates with two dimers of histones H2A and H2B to form the nucleosome that functions to bind and compact DNA [21]. Core histones H3 and H4 are rarely found as subunits in protein complexes other than nucleosomes or nucleosome variants. A few examples of H3/H4 interacting proteins include chaperones anti-silencing function protein 1 (Asf1) and nuclear autoantigen sperm protein (NASP) [22,23]. However, their interaction is more dynamic given the roles of Asf1 and NASP in nucleosome assembly, as chaperones need to bind and release histones H3 and H4 [24]. To our knowledge, the Lamin B receptor–histone–heterochromatin protein 1 complex, which helps anchor heterochromatin to the nuclear envelope, is one of the only examples of a complex that stably associates with histones H3 and H4 [25].

A previous histone H3 depletion study in yeast cells showed reduced rates of rRNA synthesis, suggesting that histone H3 is required for UAF to activate Pol I transcription [26]. Histone H3 depletion also caused a decrease in Rrn5 protein levels, suggesting that histone H3 may play an important role in maintaining UAF complex stability, and is possibly required for UAF assembly and complex integrity [26]. These results are specific to histone H3, as a similar histone H4 depletion study led to no detectable changes in rRNA synthesis [27].

Like CF, UAF is another Pol I initiation factor that is expressed at very low levels and is likely one of the least expressed among all the Pol I factors [28]. UAF isolation from yeast requires an extensive purification and concentration scheme that only achieves modest quantities of UAF sufficient for activity assays, but not for higher end biochemical and structural studies [12,13]. To overcome this challenge, we developed a recombinant system to express and purify transcriptionally active UAF from *Escherichia coli*. Using our recombinant system, we explored the roles of histones H3 and H4 in UAF complex assembly and integrity, and determined the stoichiometry of the histone subunits in the complex. Together, our results provide evidence that UAF contains an H3–H4 tetramer-like subcomplex.

## Results

### Expression and purification of recombinant UAF

**Fig. 1.**
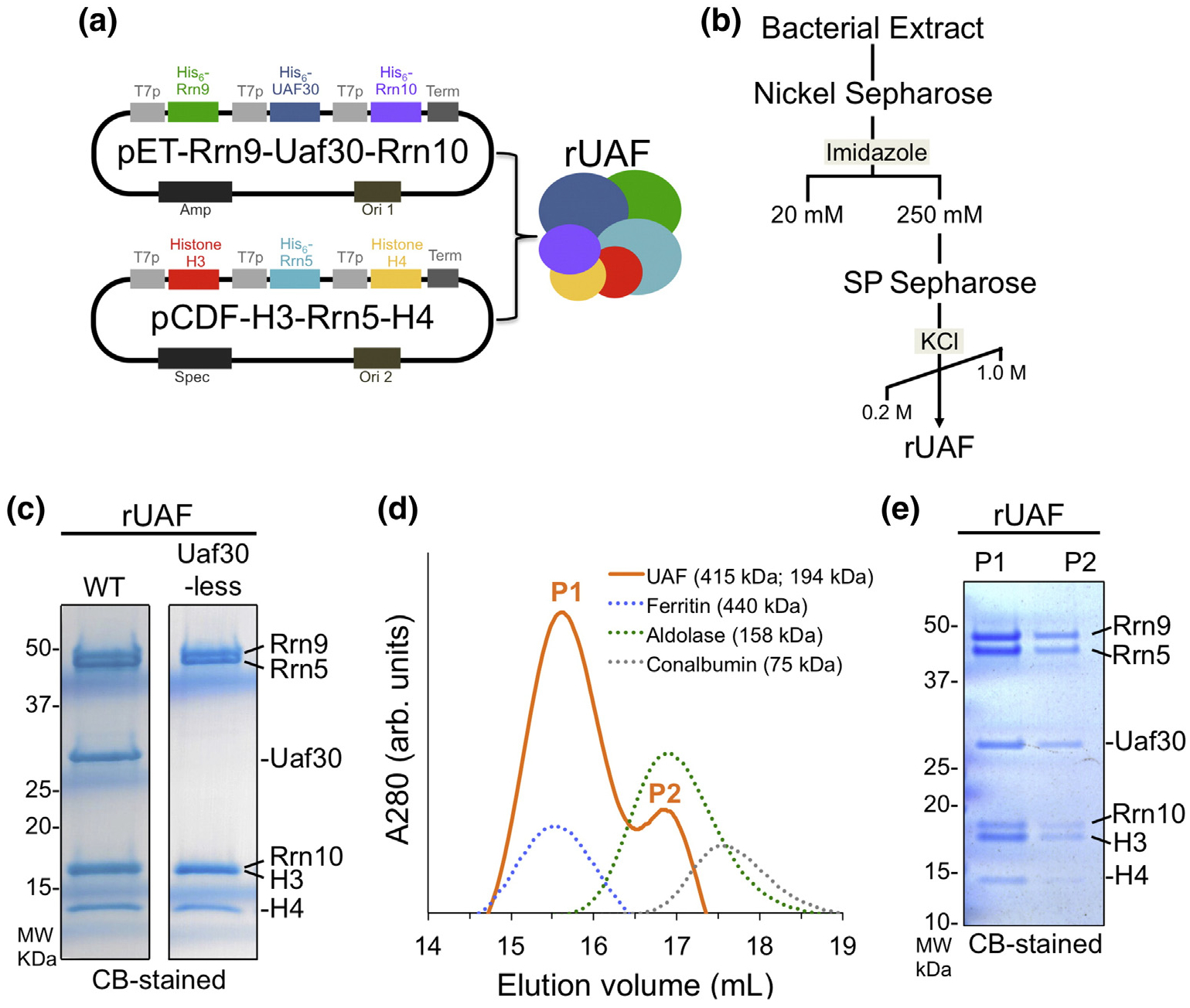
Expression and purification of rUAF. (a) Diagram of the two T7 bacteria expression vectors used to coexpress rUAF complex. T7P, T7 promoter; Term, T7 terminator; amp, ampicillin; spec, spectinomycin; ori, origin of replication. (b) rUAF purification scheme. (c) Coomassie blue (CB)-stained SDS-PAGE gel of purified rUAF (left) and rUAF lacking the Uaf30 subunit (right). Note that Rrn10 and histone H3 resolved as a single band on a 4%–20% Tris–glycine gel. MW, molecular weight. (d) SEC of purified rUAF. Elution profile of UAF (solid orange line) and standards (dashed line). Estimated molecular weight of UAF peaks and standards are indicated. P1/P2, peak 1/2; arb. units, arbitrary units. (e) CB-stained Bis–Tris SDS-PAGE gel of UAF peaks 1 and 2.

We developed a recombinant coexpression system to simultaneously express all six UAF subunits from two vectors in *E. coli*. We cloned three UAF genes into one of two vectors with compatible replicons and antibiotic resistance markers (Fig. 1a). Multiple UAF subunits were tagged with hexahistidine (His_6_) for enrichment by Nickel affinity chromatography and then further purified by cation exchange (Fig. 1b). This two-step purification scheme yielded pure recombinant UAF (rUAF) complex and the stoichiometry resembles that of yeast isolated UAF (Fig. 1c). For instance, Rrn5 and Rrn9 consistently appear as a stoichiometric doublet and the intensity of histone H4 is less than histone H3 [12,13]. We used a similar coexpression and purification system to purify the UAF complex without the Uaf30 subunit. Yeast isolated UAF does not require Uaf30 to maintain complex integrity [14], and similar to yeast, Uaf30-less rUAF forms a stable complex (Fig. 1c).

To confirm that each subunit is in a single complex, UAF was further purified by high-molecular-weight cutoff filtration followed by size-exclusion chromatography (SEC). Initially, UAF eluted reproducibly later than expected compared to known molecular weight markers (data not shown), suggesting that it was non-specifically binding to the SEC column. Increasing the salt concentration and adding arginine to the SEC column buffers prevented non-specific binding to the column. Using these conditions in subsequent SEC experiments, rUAF eluted as two single peaks between standards, Ferritin (440 kDa) and Aldolase (158 kDa) (Fig. 1d). The equal staining of each UAF subunit in both peaks suggests that the first peak contains UAF homodimers and the second, later eluting, peak contains UAF monomers (Fig. 1e).

Given the low yields of yeast UAF in the extract, we were unable to precisely determine the apparent molecular weight. The theoretical molecular weight of UAF, assuming each subunit is in single copy, is 159.2 kDa as a monomer and 318.4 kDa as a dimer. Using the retention volume of both UAF peaks and multiple SEC standards, the apparent molecular weight of UAF homodimer is ~415 kDa and monomer is ~194 kDa. However, the apparent molecular weight of the rUAF monomer estimated here is larger than the theoretical molecular weight of UAF if each subunit is in a single copy in the complex. The difference between the theoretical MW and our calculated apparent MW is ~35 kDa, suggesting that there may be multiple copies of one or more UAF subunits.

The apparent molecular weight of rUAF is also roughly consistent with yeast isolated UAF, which was previously shown to elute as a single peak between size markers ß-amylase (206 kDa) and BSA (66 kDa) [12]. To directly compare the SEC elution profile of rUAF with native yeast UAF complex, we separated a yeast extract that expresses a C-terminal 3X–FLAG--tagged Rrn5 in parallel with our rUAF. We found that yeast and rUAF yielded a similar elution profile (Fig. S1A, B), further supporting the conclusion that our recombinant system produces a stable UAF complex with similar biochemical properties to the yeast UAF complex.

### DNA binding and transcriptional activity of rUAF

A major function of UAF is to interact with the UAS element in the Pol I promoter DNA. To test if our rUAF binds DNA, we used an *in vitro* promoter pulldown assay where we conjugated biotinylated Pol I promoter DNA containing the UAS element to streptavidin beads. We incubated rUAF with the Pol I promoter bound beads and observed a ~5-fold enrichment of rUAF binding over beads alone lacking DNA (Fig. S2A, B). Together, these results show that our rUAF possesses the ability to bind DNA as would be expected for yeast UAF.

**Fig. 2.**
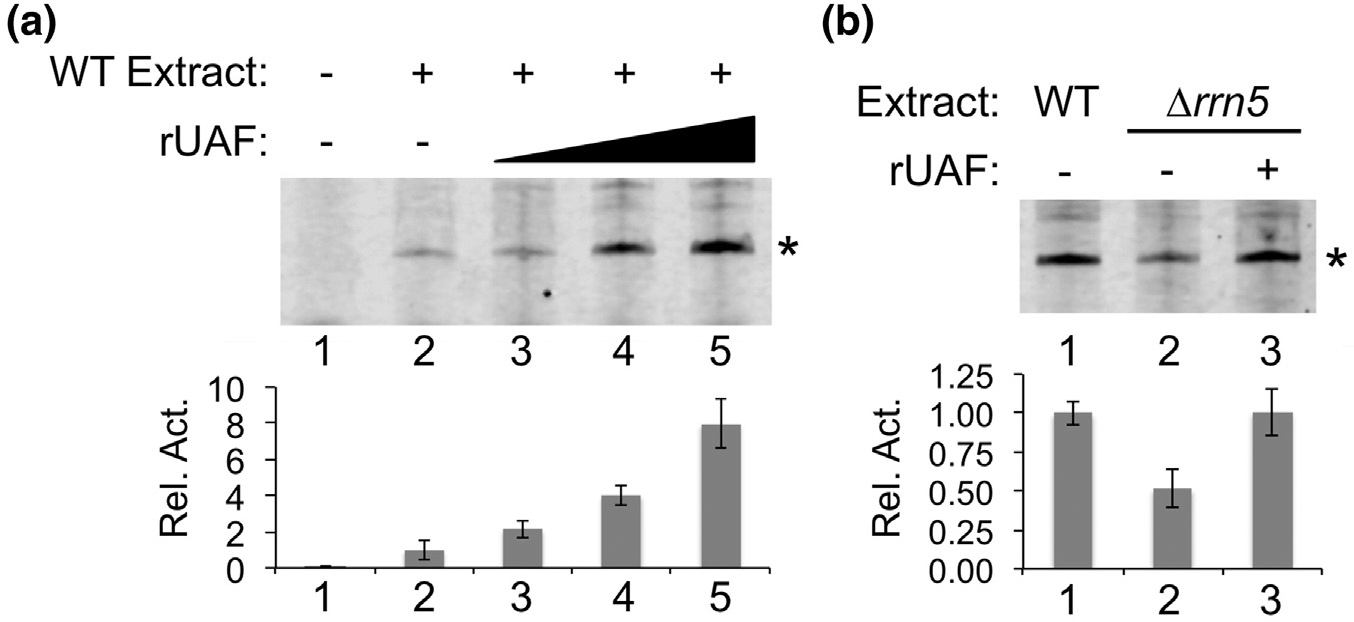
Transcription activity of rUAF. (a). Increasing amounts of rUAF (5, 10, and 20 nM) were added to transcription competent wild-type (WT) yeast extracts. Pol I primer extension products were analyzed by a Urea containing SDS-PAGE gel. (b). rUAF (20 nM) was added to Δ*rrn5* transcription competent yeast extracts. Activity assays were performed in duplicate and representative assay results are shown. Primer extension products are denoted with an asterisk to the right of the gel image. Band intensities of primer extension products are depicted below the gel images as a bar graph. Relative activities (Rel. Act.) were normalized to the activity in absence of rUAF, which was set at 1.0. Error bars denote standard deviation. Transcription activity assays were performed in duplicate and representative assay results are shown. Error bars denote standard deviation.

Previous studies show that the addition of yeast purified UAF stimulates Pol I activity of transcription--competent wild-type extracts *in vitro* [12]. Likewise, the addition of rUAF to wild-type extracts also stimulated transcriptional activity (Fig. 2a). To ensure the stimulatory activity was UAF dependent, we performed a similar transcription assay using an *rrn5*Δ extract. As expected, transcriptional activity is reduced in the *rrn5*Δ extract compared to a wild-type extract, and the addition of rUAF restores the level of transcriptional activity back to wild-type levels (Fig. 2b). From these results, we conclude that rUAF is transcriptionally active and stimulates Pol I activity as previously shown for yeast purified UAF.

### UAF complex formation in the absence of individual UAF-specific subunits

**Fig. 3.**
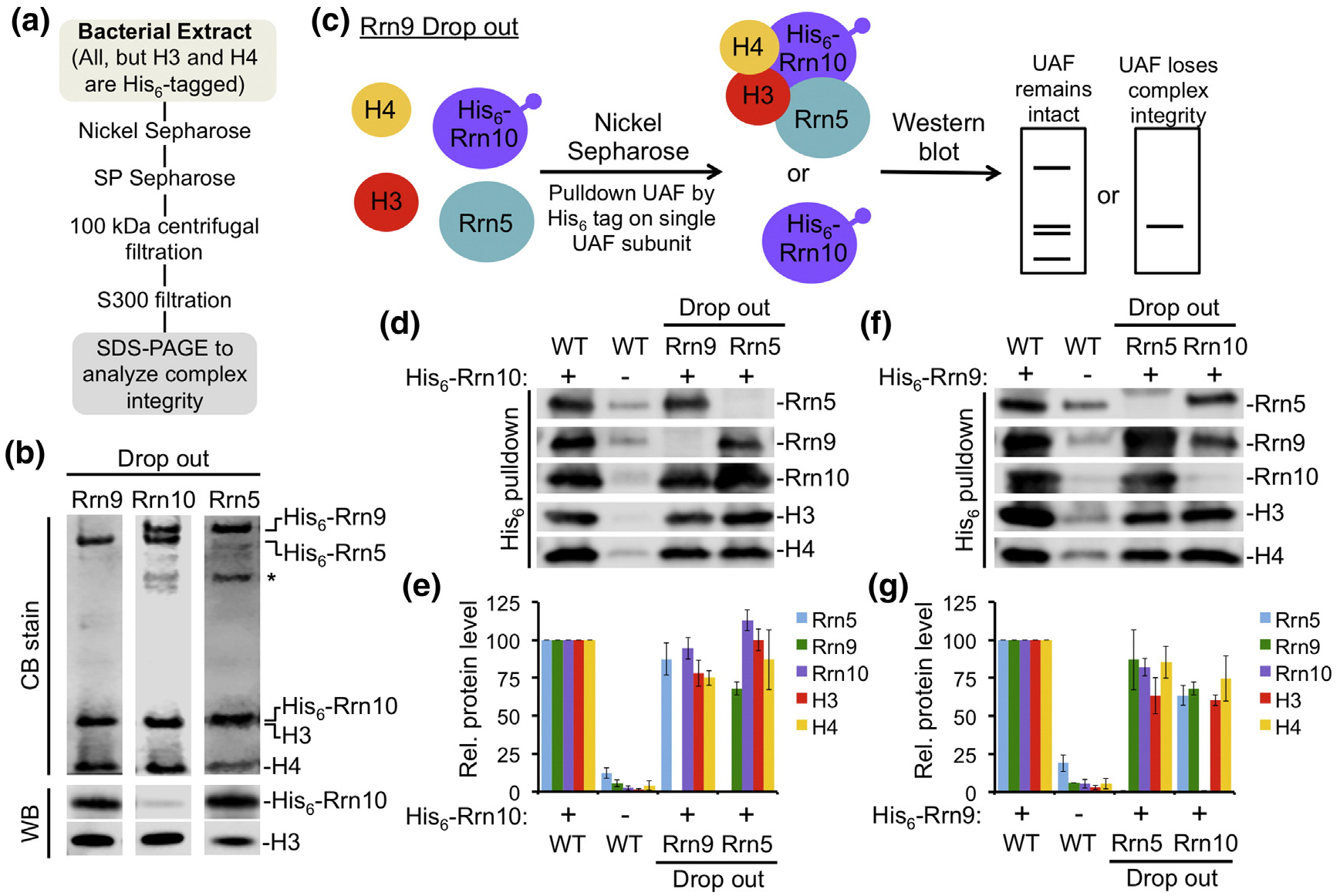
UAF complex integrity is not dependent on a single UAF-specific subunit. (a) Purification scheme and (b) SDS-PAGE analysis of rUAF complexes lacking the Rrn9 subunit, the Rrn10 subunit, or the Rrn5 subunit. Coomassie blue (CB) staining is shown above. Western blot (WB) analysis against Rrn10 and histone H3 is shown below. An asterisk denotes partial degradation products of Rrn9. (c). Schematic representation of the pulldown method used to analyze UAF complex integrity in the absence of the Rrn9 subunit. A similar strategy was used to determine UAF complex integrity in the absence of the Rrn10 subunit or the Rrn5 subunit. Western blot analysis of isolated UAF complexes pulled down by His_6_-tagged Rrn10 (d) or His_6_–Rrn9 (f). UAF was pulled down in the presence of all subunits (WT), without the Rrn9 subunit (Rrn9 drop out), without the Rrn5 subunit (Rrn5 drop out), or without the Rrn10 subunit (Rrn10 drop out). (e, g). Relative (Rel.) protein levels of the drop out complexes were normalized against WT, which was set at 100. Pulldown assays were performed in duplicate and representative assay results are shown. Error bars denote standard deviation.

Although a partial UAF complex can still form in the absence of Uaf30, it is unclear whether a complex can still form in the absence of the other subunits. We used our rUAF expression system to examine complex formation in the absence of Rrn5, Rrn9, or Rrn10 (Fig. 3a). In our coexpressions, we also excluded Uaf30 since it is dispensable for complex integrity as shown above. We expressed and purified these complexes by Nickel affinity and cation exchange chromatography followed by concentration on a 100-kDa cutoff filter and mini S200 cartridge. In the absence of Rrn5, Rrn10, or Rrn9, all the remaining UAF subunits co-purified (Fig. 3b), indicating that none of the UAF specific subunits are essential for complex integrity. We note that in the absence of Rrn5, we observe slightly reduced levels of histones H3 and H4, possibly suggesting that Rrn5 may play a role in UAF complex stability at the level of histone subunit interaction. However, we also note that in the absence of Rrn5 and in the absence of Rrn10 to a lesser extent, Rrn9 partially degrades during the expression and purification process in a reproducible manner (Fig. 3b, asterisk), which could potentially contribute to the modest reduction in histone subunits. Furthermore, in the absence of Rrn5, Rrn10, and Rrn9, we notice a reduction in the overall yield of UAF compared to expression when none of these subunits are absent. Ultimately, in terms of rUAF expression, this further emphasizes the importance of coexpressing the entire complex in order to achieve intact UAF complex.

Since multiple UAF subunits were His_6_-tagged, we used a similar coexpression strategy above, but placed a His_6_-tag on only one of the UAF subunits (Fig. 3c). In agreement with our results above, all the remaining UAF subunits co-purified with His_6_-Rrn10 in the absence of Rrn5 or Rrn9 (Fig. 3d, e). Likewise, all the remaining UAF subunits co-purified with His_6_–Rrn9 in the absence of Rrn5 or Rrn10 (Fig. 3f, g), further suggesting that no single UAF specific subunit is required for complex integrity. Overall, these pulldown studies show that several subcomplexes can still form when each UAF-specific subunit is absent.

### Interactions between histone H3 and the UAF-specific subunits

**Fig. 4.**
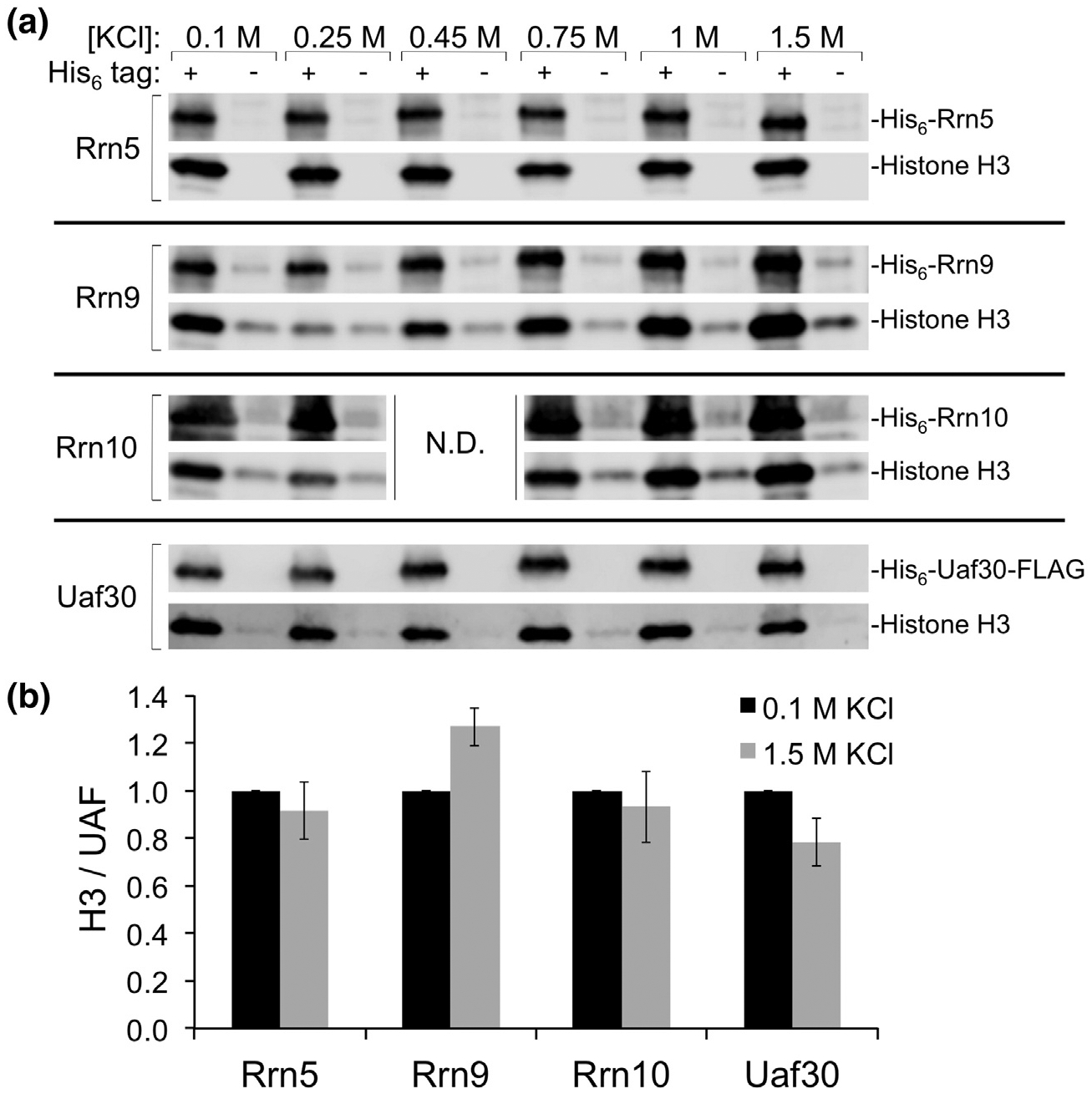
Effect of increasing ionic strength on H3 interaction with each UAF-specific subunit. (a) Histone H3 and individual UAF-specific subunits with (+) or without (−) a His_6_-tag were coexpressed in *E. coli* and purified by Nickel affinity. Precipitated complexes were split evenly and washed with the indicated concentrations of potassium chloride (KCl) before elution from beads. The elutions were then analyzed by SDS-PAGE and Western blot. N.D., not determined. (b) Pulldown assays for the 0.1 M and 1.5 M KCl conditions were performed in duplicate. Relative (Rel.) protein levels of the pulldowns were normalized against WT as the ratio of histone H3 over the indicated UAF--specific subunit, which was set at 1.0. Error bars denote standard deviation.

It is unclear how histone H3 and/or H4 interact with multiple UAF-specific subunits. For instance, a subset of UAF-specific subunits could interact exclusively with H3, while others interact with histone H4. To differentiate between these possibilities, we performed Nickel affinity pulldowns with individual His_6_-tagged UAF-specific subunits and either histone H3 or H4. Given the insolubility of histone H4 in the absence of H3, we were unable to test H4 alone as it was consistently insoluble. When we coexpressed histone H3 with individual His_6_-tagged UAF-specific subunits under various conditions, we found that each subunit interacted with histone H3 (Figs. 4, S3, S4), suggesting that H3 directly interacts with each UAF-specific subunit in the absence of H4, and H3 may be responsible for connecting H4 to the UAF complex. To test the stringency of our protein–protein interactions, we tested the effect of increasing ionic strength and detergent in our binding assays. After incubating our coexpressed proteins with the Nickel Sepharose beads, we split the beads equally into separate tubes and then washed the beads with buffers containing a range of salt and detergent concentrations from low to high. We found that histone H3 still co-purified with each UAF-specific subunit independent of the ionic strength or percent detergent in our buffers (Figs. 4a–d and S3A–D), suggesting that histone H3 stably interacts with each UAF-specific subunit.

Since histone H3 can bind DNA and the UAF complex is a DNA-binding factor, we reasoned that DNA, or possibly RNA, could co-purify in our pulldown assays and mediate an indirect protein–protein interaction. To test this, we performed our pulldowns in the presence of ethidium bromide (EtBr), RNase A, and DNase I. Each UAF-specific subunit still co-purified histone H3 following EtBr treatment, or digestion with RNase A or DNase I (Fig. S4A–D), suggesting that histone H3 binding to each UAF-specific subunit is not dependent on the presence of DNA or RNA. However, we cannot exclude the possibility that DNA is protected within a shared interface between the interacting proteins.

### Histone H3 domains important for UAF subunit interaction

Core histone proteins contain two domains, a histone-fold containing body domain and an unstructured tail domain [29,30]. The histone body domains and the tail domains interact with other proteins to help facilitate transcriptional changes and other processes at the chromatin level. To determine the importance of the histone H3 tail and/or body domains for UAF-specific subunit interactions, we performed pulldowns with each His_6_-tagged UAF-specific subunit and a tailless histone H3 mutant. Using a similar coexpression and pulldown approach described above, we detected reduced tailless histone H3 binding to Rrn5 (Fig. S5A, E), indicating that the tail domain helps mediate interaction with Rrn5. We also detected reduced tailless histone H3 binding to Rrn10, Rrn9, and Uaf30 compared to full-length histone H3 (Fig. S5B–E), indicating the importance of the H3 tail domain for these interactions. We still observe a much lighter, but consistent band for the tailless histone H3 in our pulldown assays for Rrn5, Rrn9, Rrn10, and Uaf30 suggesting that the body domain contributes their interaction.

Likewise, we observed reduced levels of tailless histone H3 when we assemble the UAF complex with all remaining subunits, with the exception of Uaf30 (Fig. S6A, B). This result agrees well with the pairwise interaction assays in that the histone H3 tail domain is important for UAF interaction, but the body domain is still sufficient for interaction, although at a reduced level. Taken together, these results suggest that the histone H3 body and tail domains are important for UAF-specific subunit interaction.

### UAF complex formation in the absence of histone subunits

**Fig. 5.**
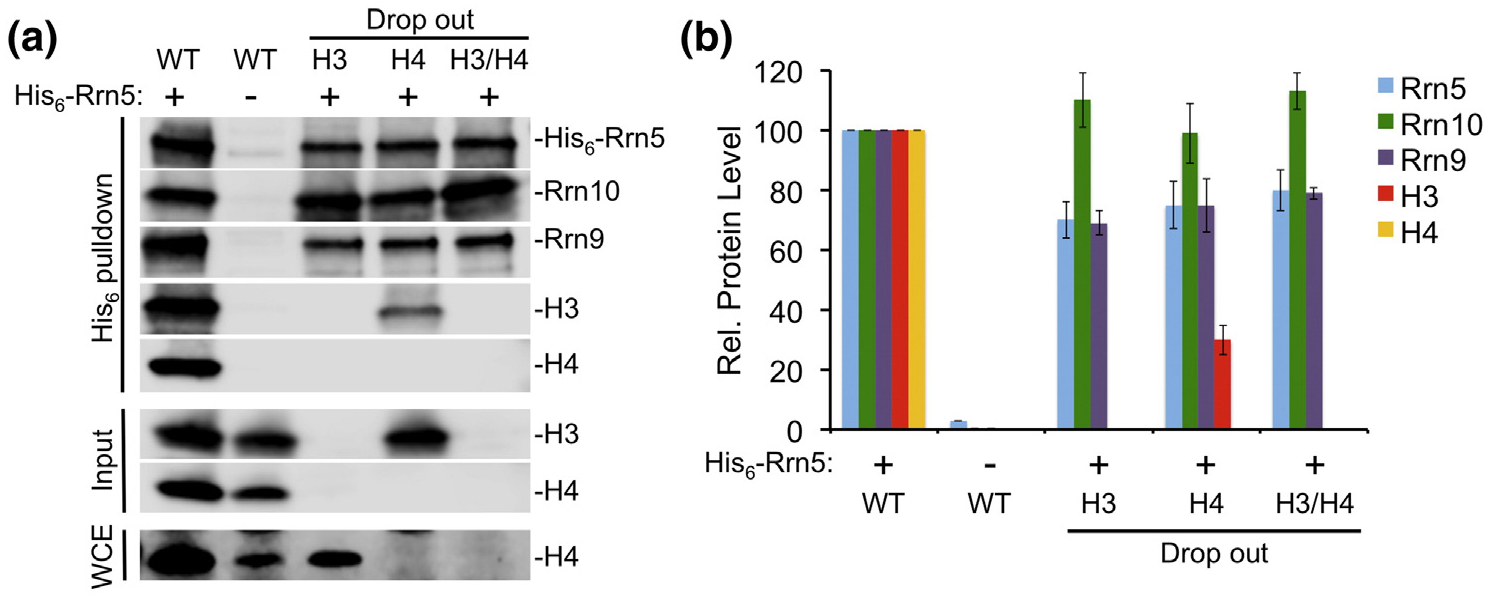
UAF-specific subunit complex formation in the absence of the histone subunits. (a) Western blot analysis of isolated UAF complexes pulled down by His_6_-tagged Rrn5. UAF was pulled down in the presence of all subunits (WT), without the histone H3 subunit (H3 drop out), without the histone H4 subunit (H4 drop out), or without both histones H3 and H4 (H3/H4 drop out). The soluble input was analyzed by SDS-PAGE and Western blot for the presence of histones H3 and H4 (Input). Whole cell extracts were also analyzed for the presence of histone H4 (WCE). (b) Relative (Rel.) protein levels of the drop out complexes were normalized against WT, which was set at 100. Pulldown assays were performed in duplicate and representative assay results are shown. Error bars denote standard deviation.

Previous studies suggest that histone H3 is required for UAF complex assembly and integrity [26]. Since histone H3 stably interacts with each UAF-specific subunit, we propose that H3 could act as a scaffold to help assemble the entire complex. To test this, we used our recombinant expression system to determine whether histones H3 and/or H4 are necessary for UAF complex assembly and integrity. We created UAF coexpression vectors that contain a single His_6_-tag on the N-terminus of Rrn5. When all UAF subunits are coexpressed, each subunit is pulled down with His_6_–Rrn5 demonstrating that this approach makes a stable UAF complex (Fig. 5a, b). We repeated the Nickel affinity pulldown using coexpression vectors where one or both of the histone genes were removed. Strikingly, in the absence of both histones H3 and H4, the remaining UAF subunits were still able to form a complex (Fig. 5a, b). This challenges the previous model that the histones are required for complex assembly, and supports a new model where the histone subunits are not required for UAF complex integrity.

As expected from the histone binding studies above, histone H3 still associates, although at a reduced level, with the partial UAF complex in the absence of histone H4 (Fig. 5a, b). This may indicate that histone H3 stoichiometry is dependent on histone H4. In the absence of histone H3, we could not detect association of histone H4 with the other UAF subunits (Fig. 5a, b). This is likely due to the insolubility of histone H4 in the absence of H3 described above since we could only detect H4 in the whole cell extract (Fig. 5a). From these results, we speculate that histone H4 may not make contacts with other UAF-specific subunits independent of histone H3, as we would expect the UAF-specific subunits to help solubilize histone H4. However, we cannot rule out the possibility that histone H4 makes contact with the UAF-specific subunits when it is in the soluble fraction.

### Histone subunit stoichiometry in UAF

Our SEC results suggest that there may be more than one copy of multiple UAF subunits. Consistent with this idea is the observation that histone H3 stains more intensely in our rUAF preparations *versus* the lighter staining histone H4 band, which is also observed in yeast UAF preparations [13]. It is plausible that UAF contains a second copy of histone H3 as this would help facilitate interactions with multiple UAF subunits. This possibility aligns well with the reduction of histone H3 association in the absence of histone H4, as H4 may aid in the binding of a second histone H3 subunit. Taken together, we hypothesize that UAF contains more copies of histone H3 than histone H4.

**Fig. 6.**
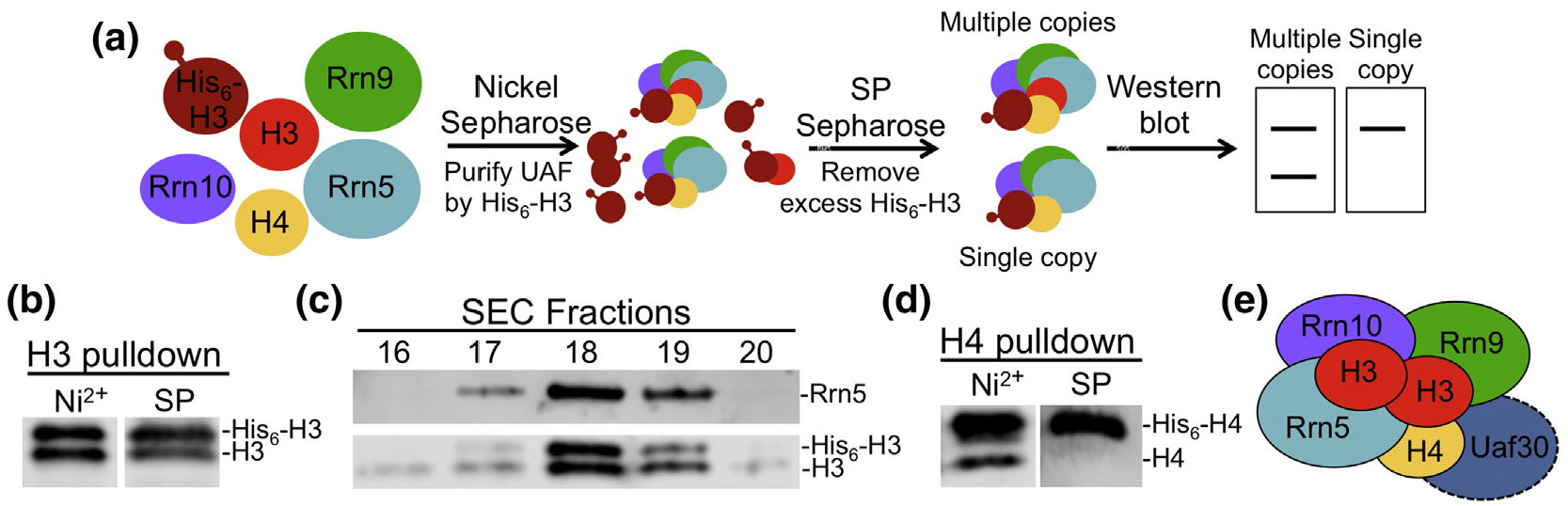
Stoichiometry of histone H3 and H4 subunits in UAF. (a). Schematic representation of the pulldown method used to determine histone H3 stoichiometry. UAF subunits including both a tagged and nontagged version of histone H3 were coexpressed and purified by Nickel sepharose followed by SP sepharose. Western blot analysis was then used to determine if both the tagged and nontagged forms of H3 were present in purified UAF. Western blot analysis of the Nickel sepharose and SP sepharose elutions of (b) H3 pulldowns and (d) H4 pulldowns probed using anti-histone H3 or H4 antibodies (c). UAF SEC fractions were analyzed by Western blot using antibodies against Rrn5 and histone H3 (e). UAF forms a stable complex with two copies of histone H3, and one copy of histone H4. These histone proteins combine with the putative histone fold domain of Rrn5 to form a hybrid tetramer-like core complex. The remaining UAF-specific subunits, Rrn10, Rrn9, and Uaf30 envelop the tetramer-like core complex.

We developed a UAF expression strategy to test if there is more than one copy of histone H3 in the complex. To this end, we coexpressed non-tagged versions of the UAF subunits on two compatible expression vectors as done previously, but included a third compatible expression vector with His_6_--tagged histone H3 that is used to isolate the complex (Fig. 6a). The tagged and nontagged versions of histone H3 are different molecular weights, so we can easily distinguish them by Western blot using an H3-specific antibody. After coexpression, we purified the complex by Nickel affinity and cation exchange chromatography, and used Western blot to determine if nontagged histone H3 copurified with tagged H3. Our results showed two histone H3 bands throughout each purification step (Fig. 6b), suggesting that UAF contains multiple copies of histone H3. To ensure that the two histone H3 bands were not due to a mixed population of UAF dimers, we used SEC and Western blot to determine if the monomer UAF fraction also showed two bands for histone H3. Both the nontagged and tagged versions of histone H3 were present in the monomer fraction, demonstrating that UAF contains at least two copies of histone H3 (Fig. 6c).

We used a similar strategy to determine the stoichiometry of histone H4. We expressed UAF with nontagged and His_6_-tagged versions of histone H4, purified the complex as described above, and used Western blot to determine if the UAF complex contained both versions of H4. Both the tagged and nontagged versions of histone H4 were in the Nickel affinity elutions, but only tagged histone H4 remained after further purification by cation exchange (Fig. 6d). The loss of the nontagged histone H4 suggests that there is a single copy of H4 in the UAF complex.

**Fig. 7.**
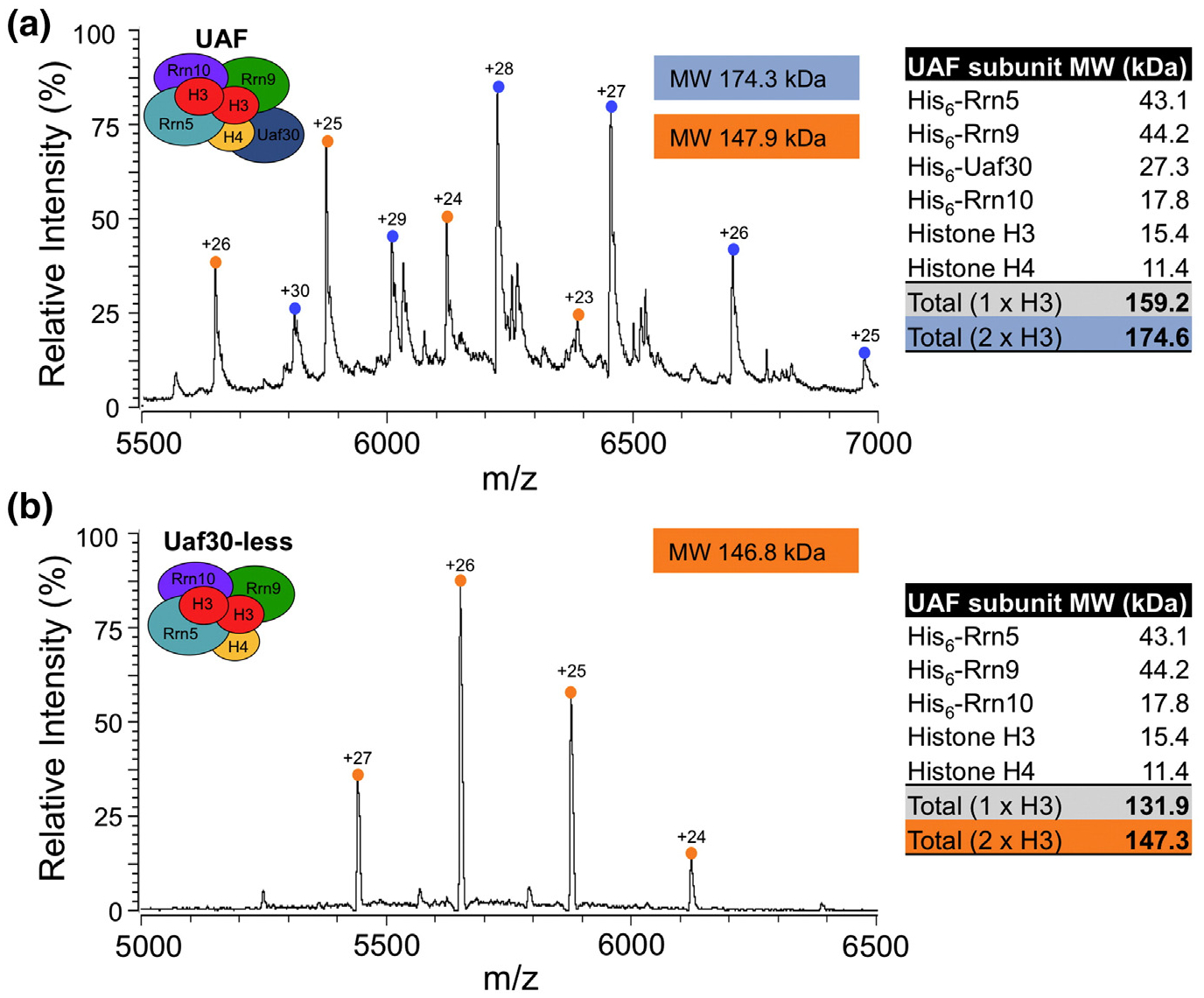
ESI-MS analysis of UAF. Native mass spectra of UAF (a) or Uaf30-less UAF (b) are shown. The UAF and Uaf30-less UAF charge state distributions are noted with blue and orange circles, respectively. The charge states are listed above their corresponding peaks. The deconvoluted molecular weights (kDa) of the complexes are shown as light blue and orange colored boxes within the mass spectrum. The theoretical molecular weights of each UAF subunit and complexes containing single or two copies of histone H3 are listed in a table to the right of the mass spectra.

Next, we used Native mass spectrometry (MS) to determine the precise stoichiometry of the UAF subunits in the complex. For Native-MS, we analyzed rUAF after SP Sepharose purification, which contains a mixture of monomers and dimers. The sum of the theoretical molecular masses of each UAF subunit as predicted from their coding sequences in the coexpression vectors is 159.2 kDa, assuming the stoichiometry of the subunits with respect to each other is 1:1 (Fig. 7a). The spectrum reveals two prominent charge state distributions centered around *m*/*z* 6230 and 5880 (Fig. 7a). The mass determination of the ion series with the highest *m*/*z* values yielded a molecular mass of 174.3 kDa, a 15.1 kDa increase in molecular mass which is nearly identical to the molecular mass of histone H3 of 15.4 kDa. We note that we did not observe any UAF dimers by Native-MS, which may be due to the volatile buffer conditions used to prepare the samples that could disrupt self-association. Combined with our results above, we conclude with high confidence that the UAF complex contains two copies of histone H3 and the remaining UAF subunits are in single copies.

The mass determination of the second ion series yielded a molecular mass of 147.9 kDa (Fig. 7a). The closest theoretical mass matching this experimentally determined molecular mass is 147.3 kDa corresponding to a UAF complex containing two copies of histone H3 but lacking the Uaf30 subunit. Since the Uaf30 subunit is dispensable for UAF complex integrity, this supports our model that Uaf30 may lie at the periphery of the complex and could more easily disassociate compared to the other UAF subunits. To further confirm this, we analyzed our UAF complex lacking Uaf30 by Native-MS. We observed a single ion series centered around *m*/*z* 5660 that yielded an experimental molecular mass of 146.8 kDa (Fig. 7b), which is in near-perfect agreement with the theoretical molecular mass of UAF that contains two copies of histone H3 and lacks Uaf30 (Fig. 7b). Together, our stoichiometry results support a model that UAF contains a trimer of histone subunits that include a single copy of histone H4 and two copies of histone H3.

## Discussion

Here, we developed a robust recombinant system to express high microgram quantities of transcriptionally active UAF. We used this system to understand the contribution of the histone H3 and H4 subunits in UAF complex assembly, and to understand how histone H3 associates with the UAF-specific subunits. We note that rigorous testing of UAF transcriptional activity remains a work in progress and the activity of the histone-free subassemblies remains to be determined. We anticipate that this system will help facilitate future functional, biochemical, and structural studies of the UAF complex that is one of the last remaining pieces of the yeast Pol I transcription initiation puzzle.

Our SEC studies of UAF showed the formation of a homodimer in solution. Pol I factor dimerization is not unique to UAF as previous studies have shown that CF (unpublished observation), Rrn3, TBP, and Pol I form stable dimers in solution [31–34]. There is also evidence that shows both TBP and Pol I form dimers *in vivo* [35,36]. Current regulatory models suggest that Pol I and Rrn3 dimers are transcriptionally inactive as they would be unable to interact with rDNA, and this may serve as a regulatory mechanism for Pol I transcription [35,37,38]. Interestingly, the mammalian Pol I activator UBF binds the upstream element of the human Pol I promoter as a dimer [39–41]. Further investigation is needed to determine if UAF homodimerization can be detected *in vivo* or whether it is an *in vitro* byproduct of highly pure and concentrated rUAF complex.

Histones H3 and H4 are essential for yeast growth, making it difficult to understand what functional role histone H3 and H4 may play in the UAF complex [42]. Previous studies using an *in vivo* histone H3 depletion system show that the partial depletion of histone H3 leads to a large decrease in Pol I transcription and causes a decrease in UAF stability [26]. These findings suggest that histone H3 is required for UAF complex integrity and assembly. However, this study is confounded by the numerous consequences that occur when histone H3 is depleted *in vivo*. For instance, H3 depletion in *S. cerevisiae* led to ~2500 genes to lose repression at the promoter due to ~2000 mislocalized nucleosomes [43]. Given that nearly half of the yeast genome becomes misregulated when H3 is depleted, it is unclear if the decrease in rRNA synthesis is an indirect effect of H3 depletion rather than a direct loss of H3 from the UAF complex. Using our recombinant system, we were able to directly test the importance of histones H3 and H4 in UAF complex formation. Intriguingly, we found that histones H3 and H4 are not required for UAF complex formation as Rrn5, Rrn9, and Rrn10 were still able to form a complex in the absence of the histone subunits. It still remains unclear how UAF may acquire histone subunits in the complex. For instance, the UAF-specific subcomplex could interact with a nucleosome and evict H2A and H2B, as they are not present in the UAF complex [13]. Likewise, the UAF-specific subcomplex could associate with non-chromatin associated histones, in the form of H3–H4 tetramers or dimers as they are transported to the nucleus *via* various chaperones [22,24,44]. It is also unclear how a single copy of histone H4 would become lost during this process. Either way, additional studies will be necessary to elucidate the mechanism for how UAF acquires the histone subunits.

Our studies demonstrate that there are two copies of histone H3 and a single copy of histone H4 in the UAF complex. Histones are most commonly found in pairs, suggesting that UAF may contain an additional histone-like subunit and/or domain. We hypothesize that a UAF subunit may replace the missing histone H4. Consistent with this, preliminary bioinformatic findings revealed the possibility that Rrn5 may contain a histone fold domain that could pair with the additional copy of histone H3 [45]. Therefore, in combination with our interaction studies, we propose an attractive, although speculative, model suggesting that UAF contains a hybrid H3–H4 tetramer-like subdomain composed of two copies of histone H3, one copy of histone H4, and a second histone-like domain of Rrn5 (Fig. 6e). It is presently unclear if Rrn5 is most closely related to histone H4 given the remote homology, and more detailed studies will be necessary to understand its importance in UAF complex assembly and function.

In comparison to other histone fold domain-containing complexes such as TFIID [46–50], yeast Hap2/3/5 [51–53] and its mammalian ortholog NF-Y [54,55], SAGA [48,50,56–58], and yNC2 [59,60], our model would suggest that UAF lies between the histone octamer-like complexes of TFIID and SAGA and the dimer-like complexes of Hap2/3/5 and yNC2. Our model also has implications for how UAF may interact with the UAS element. The tetramer-like subcomplex of UAF could potentially wrap DNA around itself once in a tetrasome-like manner, forming a horseshoe-shaped DNA structure [61,62]. Based on the presence of histones H3 and H4 in the complex, it was previously speculated that UAF may wrap DNA around itself twice like a nucleosome [13,18]. The UAS promoter element is ~95 bp in length based on transcription activity assays [15–17], while UAF footprinting to the Pol I promoter *in vitro* only protects 59 bp within the UAS [63]. Together, these boundaries are more in line with a tetrasome-like structure that occupies ~80 bp of DNA [64,65] rather than the 147 bp bound and protected by a nucleosome.

Rrn5 also contains a SANT domain [66,67]. SANT domains are broadly found in chromatin remodeling enzyme complexes [68–70] and have been shown to interact with various types of histone tails as well as DNA [69,71,72]. The Rrn5 histone fold domain could mediate its interaction with the histone H3 body domain, while the SANT domain could bind to the H3 tail domain. This could explain the role of both the H3 tail and body domains in binding to Rrn5. Preferential binding of the Rrn5 SANT domain to a modified or unmodified H3 tail could be a mechanism for complex assembly to regulate Pol I transcription in response to changes in cellular environment. However, it is still unclear how the remaining UAF-specific subunits contribute to histone H3 tail binding as they appear to lack a histone H3 tail binding domain as shown for Rrn5. Histone tail modifications can affect both protein and DNA interactions. For example, histone tail acetylation alters a nucleosome’s affinity for DNA, which opens the chromatin structure [21,73]. It is unclear how histone tail post-translational modifications (PTMs) could alter UAF activity, but additional studies will be necessary to determine if the histone subunits of UAF are modified by PTMs and whether PTMs affect UAF activity. We anticipate that our recombinant system will help answer these and future questions about structure, function, and regulation of UAF in the activation of Pol I transcription.

## Materials and Methods

### UAF expression and purification

Expression and purification strategies were adapted from Knutson et al. [9] and Keener et al. [13]. The genes for all six UAF subunits, *RRN9*, *UAF30*, *RRN10*, *HHT1* (histone H3), *RRN5*, and *HHF1* (histone H4) were cloned into *E. coli* expression vectors using QuikChange mega primer cloning. *RRN9*, *UAF30*, and *RRN10*, were PCR amplified and cloned into pET-Duet (Novagen). *HHT1*, *RRN5*, and *HHF1* were PCR amplified and cloned into pCDF-Duet (Novagen). The His_6_ epitope sequence was fused in frame to the 5′ ends of all UAF subunits with the exception of histones H3 and H4.

UAF was expressed by autoinduction in BL21 (DE3) RIL cells (Stratagene) or LOBSTR BL21 (DE3) RIL cells (Kerafast) in TB media (0.024% yeast extract, 0.012% tryptone, and 0.4% glycerol) supplemented with autobase (0.17 M KH_2_PO_4_ and 0.72 M K_2_HPO_4_), 5052 (0.1% alpha-lactose monohydrate, 0.25% glycerol, and 0.025% glucose), and MgSO_4_ (2 mM). After inoculation, cells were grown at 37 °C until OD_600_ ~0.5, then cooled on ice, and grown at 24 °C for 18 h. Cells were harvested by centrifugation and washed once with rUAF extraction buffer [200 mM Tris–HCl (pH 8.0), 400 mM (NH_4_)_2_SO_4_, 20 mM imidazole, 0.1% Tween-20, and 10% glycerol] supplemented with protease inhibitors and 1 mM TCEP. Pellets were resuspended in extraction buffer supplemented with 1 mg/ml lysozyme on ice for 30 min and then lyzed by sonication. Lysates were cleared and incubated with Nickel Sepharose beads (GE Healthcare) overnight at 4 °C. UAF bound beads were washed three times with wash buffer [20 mM Tris–HCl (pH 8.0), 20% glycerol, 0.1% Tween-20, 20 mM imidazole, and 450 mM KCl]. UAF was eluted in batch with wash buffer supplemented with 250 mM imidazole. Elutions were pooled and incubated overnight with SP Sepharose beads (GE Healthcare) overnight at 4 °C. UAF bound beads were washed three times with wash buffer. UAF was eluted in batch with SP elution buffer [20 mM Tris–HCl (pH 8.0), 20% glycerol, 0.1% Tween-20, and 750 mM KCl]. UAF was filtered and concentrated on a 100-kDa cutoff centricon filter (Millipore) and stored at −80 °C. All UAF expression plasmids described in this manuscript are available upon request.

### SEC

Proteins (including standards) were purified by SEC on a Superose 6 10/300 column (GE Healthcare) equilibrated in SEC buffer [20 mM Tris–HCl (pH 8.0), 20% glycerol, 0.1% Tween-20, 450 mM KCl, 0.2 M arginine] at a flow rate of 0.5 ml/min. Size-exclusion standards (GE Healthcare) were used to calibrate the column in the same SEC buffer. Peak fractions were concentrated on a 100-kDa cutoff centricon filter (Millipore), separated on a 4%–12% Bis–Tris MES gel (Invitrogen) or 4%–20% Tris–glycine gel (Bio-Rad), and analyzed by Coomassie blue staining.

For SEC analysis of yeast UAF, 6 l of yeast cells that express C-terminally 3XFLAG tagged Rrn5 were grown to an OD_600_ 1.0 in YP medium containing 3% glucose as a carbon source. Cells were harvested by centrifugation and washed twice with cold rUAF extraction buffer supplemented with protease inhibitors and 1 mM DTT. Cell pellets were weighed and resuspended in 5 ml/g extraction buffer and then mixed with an equal volume of glass beads. Cells were broken with a Bead Mixer (Biospec) until 80% of the cells were broken as guided by visual inspection with a microscope. The broken cells were cleared by centrifugation for 3 h at 100,000*g*, and the resulting supernatant was filtered three times through a 0.2-μm syringe filter. The extract contained ~15 mg/ml protein and 500 μl was loaded on the Superose 6 10/300 column in parallel with rUAF. Yeast UAF fractions (1 ml) were precipitated in trichloroacetic acid, and the resulting protein pellets were washed with 100% acetone, dried, and resuspended in 100 μl SDS-PAGE sample buffer. Protein samples were then separated on 4%–20% Tris–glycine gel (Bio-Rad), and analyzed by Western blotting using native Rrn5 or FLAG (Sigma) antibodies.

### Immobilized Pol I promoter pulldown

Dynabeads M-280 streptavidin (Invitrogen) were incubated with biotin-conjugated Pol I promoter template at 4 fmol DNA/μg beads. The yeast Pol I promoter (−203 to +41)-conjugated beads were washed four times with 1× binding/wash buffer [10 mM Tris–Cl (pH 7.5), 1 mM EDTA, 2 M NaCl] and twice with 1× transcription buffer [20 mM Hepes–KOH (pH 7.9), 50 mM KCl, 10 mM MgCl_2_, 5 mM EGTA, 0.05 mM EDTA, 2.5 mM DTT, 0.1% NP-40, and 10% glycerol]. Two μgs of bead-DNA complex was blocked with 5% casein and 5% bovine serum albumin for 2 h and then washed four times with 1× transcription buffer. Blocked beads were incubated with 1 or 2 μg of rUAF for 2 h on ice. UAF bound beads were pulled down using a magnet stand (Bio-Rad) and washed three times with 1× transcription buffer and then resuspended in SDS-PAGE sample buffer to elute the bound proteins. Eluted proteins were analyzed by SDS-PAGE and separated on a 4%–12% Bis–Tris MES gel (Invitrogen) gel and analyzed by Coomassie staining.

### *In vitro* transcription and primer extension

Transcription-competent extracts from wild-type or a*Δrrn5* yeast strain deficient in UAF activity were prepared as previously described [9]. Pol I transcription stimulation assays were assembled with 10 μg of wild-type extract mixed with increasing amounts of rUAF (5, 10, and 20 nM). Transcription recovery assays were assembled with 20 μg of wild-type or Δ*rrn5* extract supplemented with 20 nM rUAF. rUAF used in this assay was purified by Nickel and SP Sepharose and then concentrated and desalted with a 100-kDa centrifugal filter in SP elution buffer containing 200 mM KCl. Primer extension using an infrared 700-nm wavelength probe was used to visualize Pol I transcripts that were separated on Urea–TBE gel as previously described [9].

### Nickel sepharose pulldown assays

pET and pCDF plasmids (Table S1) were co-transformed into LOBSTR BL21 (DE3) RIL cells (Kerafast) and expressed by autoinduction in TB media. After inoculation, cells were grown at 37 °C until OD_600_ ~0.5 and grown at 24 °C for 16–18 h. Cells were harvested by centrifugation, washed once with rUAF extraction buffer, and frozen at −80 °C. To perform the pulldown experiment, the cell pellets were thawed and lysed with sonication, and soluble extract was prepared as described above for UAF. Cleared lysates were incubated with Nickel Sepharose beads (GE Healthcare or G-Biosciences) overnight at 4 °C. Protein-bound beads were washed four times with wash buffer [20 mM Tris–HCl (pH 8.0), 20% glycerol, 0.1% Tween-20, and 450 mM KCl] and eluted three times with two bead volumes of elution buffer (wash buffer supplemented with 250 mM imidazole). Eluted proteins were separated on 4%–20% Tris–glycine SDS-PAGE gel (Bio-Rad) and analyzed by Western blot using the following antibodies: anti-histone H3 (Abcam, ab46765), anti-histone H4 (Abcam, ab10158), anti-FLAG (sigma, F3165), and rabbit polyclonals against Rrn10, Rrn5, or Rrn9 (Knutson lab *via* Reeder lab). All Western blots were visualized using near-infrared 700 wavelength secondary antibodies (LI-COR) against rabbit or mouse and processed on an Odyssey FC imager (LI-COR). Band intensities were determined using the Image studio software package (LI-COR).

### EtBr, RNase A, DNase I, salt, and detergent treatment

Lysates were prepared for Nickel sepharose pulldown assay as described above. Treatment with EtBr and RNase A of cleared lysates was performed as described [74]. EtBr (50 μg/ml) was added to cleared lysates followed by a 30-min incubation of the lysates on ice. Lysates were incubated with Nickel sepharose beads as described above, except that the original EtBr concentration was maintained throughout bead washing. For RNase A treatment, protein-bound beads were washed two times with wash buffer prior to RNase A treatment. After washing, protein-bound beads were resuspended in wash buffer supplemented with RNase A (80 μg/ml) and incubated at 30 °C for 1 h. For DNase I, protein-bound beads were washed two times with wash buffer prior to DNase I treatment. After washing, protein-bound beads were resuspended in wash buffer supplemented with 5 μl of TURBO DNase I (Ambion) and incubated at 30 °C for 1 h. For KCl and Tween-20 treatment, protein-bound beads were separated equally into separate tubes and washed four times with TA buffer described above with various KCl concentrations or Tween-20 percentage. After treatments, protein-bound beads were eluted as described above.

### Stoichiometry pulldown assay

pET, pCDF, and pACYC plasmids (Table S1) were co-transformed into BL21 (DE3) cells and expressed by autoinduction in TB media. After inoculation, cells were grown at 37 °C until OD_600_ ~0.5 and grown at 24 °C for 16–18 h. Cells were harvested by centrifugation, washed once with rUAF extraction buffer supplemented with 1 mM DTT and protease inhibitors, and frozen at −80 °C. Cell pellets were thawed and lyzed by sonication, and soluble extract was prepared as described above for UAF. Cleared lysates were incubated with Nickel Sepharose beads (G-Biosciences) overnight at 4 °C. UAF was eluted in batch with wash buffer supplemented with 250 mM imidazole. Elutions were pooled and incubated overnight with SP Sepharose beads (GE Healthcare) overnight at 4 °C. UAF-bound beads were washed three times with wash buffer. UAF was eluted in batch with SP elution buffer. SP elutions were pooled, filtered and concentrated on a 100-kDa cutoff centricon filter (Millipore), and loaded onto a Superose 6 10/300 column (GE Healthcare). SEC was performed as described above. Peak fractions were separated on a 4%–20% Tris–glycine SDS-PAGE gel (Bio-Rad) and analyzed by Western blot.

### Native-MS

Protein complexes were buffer exchanged in 0.5 M NH_4_OAc buffer (pH 6.9) using VIVASPIN 50-kDa MWCO filters (Fisher Scientific, Hanover Park, IL). The final concentration of protein complexes was adjusted to 10 μM. A 10-μl aliquot was loaded into an offline electrospray capillary (Thermo Electron, Madison, WI) to perform the Native-MS measurement on an Exactive Plus EMR mass spectrometer (Thermo Fisher Scientific, Bremen, Germany). The sample solution was infused in positive mode at capillary voltage 1.5–1.8 kV to the instrument with the resolving power set at 17,500. The in-source and HCD collision voltages were adjusted for desolvation of the ions. The instrument was calibrated with the clusters produced by ESI of a CsI solution. The peak picking and data processing was performed with Intact Mass software (Protein Metrics, San Carlos, CA).

## Supplementary Material

dummy_label

## Abbreviations used:

Pol I: RNA polymerase I
UAF: Upstream Activating Factor
rRNA: ribosomal RNA
TBP: TATA-binding protein
CF: Core Factor
rDNA: ribosomal DNA
UAS: upstream activating sequence
SEC: size-exclusion chromatography
MS: mass spectrometry
PTMs: post-translational modifications
