# Supplementary material for: Reconstitution of RNA Polymerase I Upstream Activating Factor and the Roles of Histones H3 and H4 in Complex Assembly
Source: J Mol Biol. Author manuscript; Available in PMC 2022 Dec 13. (PMC9746128; doi:10.1016/j.jmb.2018.01.003)
Supplement: dummy_label [file NIHMS953873-supplement-dummy_label.pdf]

Supporting information for:

Reconstitution of RNA polymerase I Upstream Activating Factor and the roles of histones H3 and H4 in complex assembly

Marissa L. Smith, Weidong Cui, Ashleigh J. Jackobel, Nancy Walker-Kopp, Bruce A. Knutson

Supplemental Figures and Table:

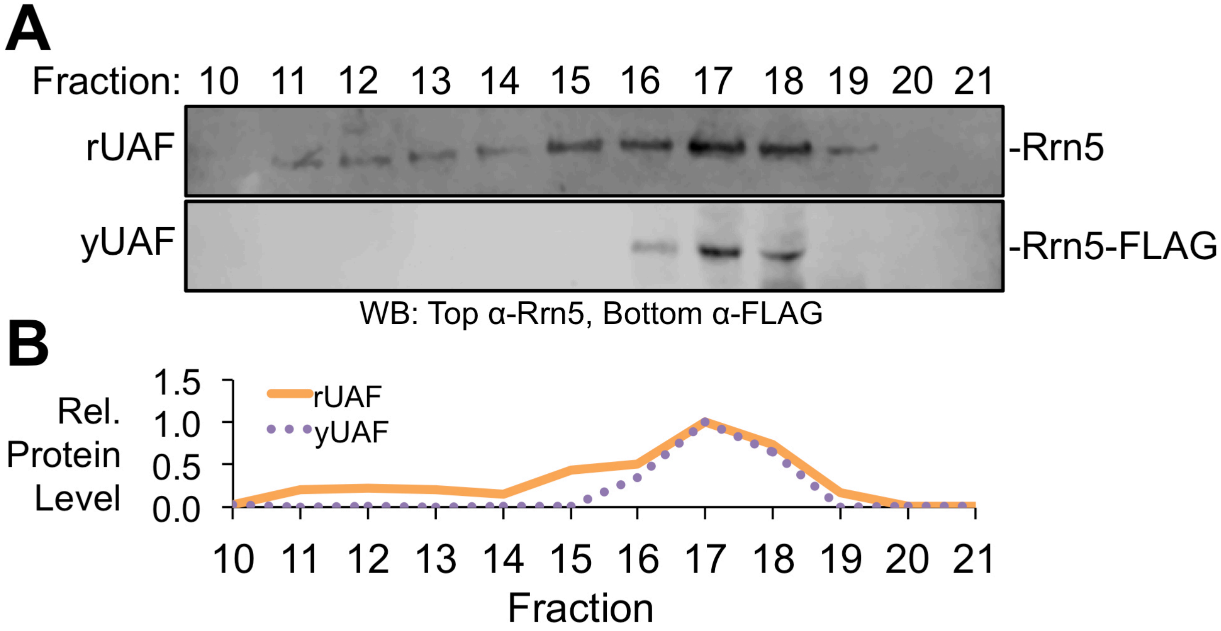

Figure S1. Comparison of recombinant and yeast UAF size exclusion elution profiles. (A). Western blot analysis of indicated size-exclusion fractions probed with either native Rrn5 or FLAG antibodies where indicated. **(B).** Relative (Rel.) protein levels of rUAF (solid orange line) and yUAF (dotted purple line) were plotted in a graph and values were normalized to the peak fraction 17, which was set at 1.0.

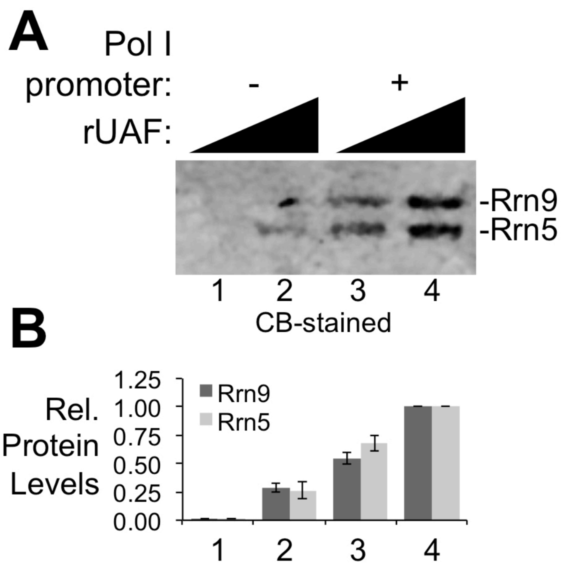

Figure S2. Pol I promoter pulldown assay of recombinant UAF. (A). Streptavidin coated beads with or without immobilized Pol I promoter DNA were incubated with 2 ug of rUAF. Beads were concentrated with a magnet, washed, and proteins were eluted and analyzed by SDS-PAGE and coomassie blue (CB) staining. **(B).** Relative (Rel.) protein levels of Rrn9 and Rrn5 were plotted in a graph and values were normalized to the highest signal, which was set at 1.0. Promoter pulldown assays were performed in duplication and errors bars denote standard deviation.

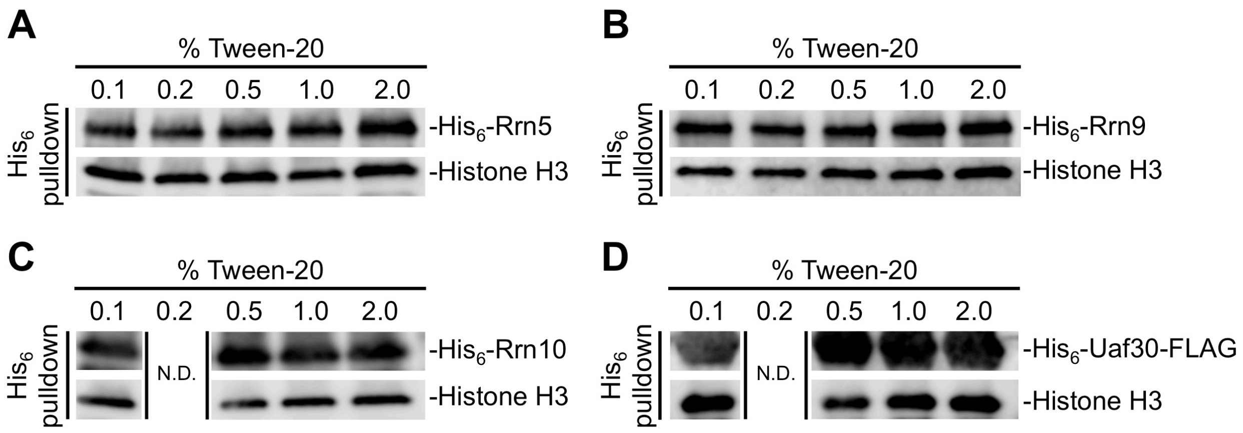

Figure S3. **Effect of increasing detergent on H3 interaction with each UAF-specific subunit.** Histone H3 and individual His_6-tagged UAF-specific subunits were coexpressed in *E. coli* and purified by Nickel affinity. Precipitated complexes were split evenly and washed with the indicated percentage of Tween-20 detergent before elution from beads. The elutions were then analyzed by SDS-PAGE and Western blot. Western blot analysis of Nickel affinity purified complexes are shown for (**A**) His_6-Rrn5, (**B**) His_6-Rrn10, (**C**) His_6-Rrn9, and (**D**) His_6-Uaf30-FLAG complexes. N.D.; Not determined.

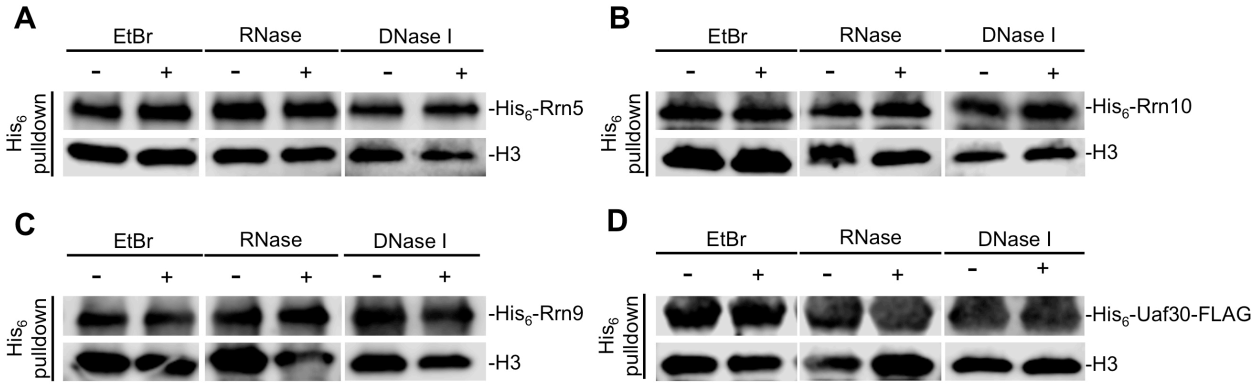

Figure S4. Effect of ethidium bromide treatment, or digestion with RNase A or DNase I on H3 interaction with each UAF-specific subunit. Each His_6-tagged UAF-specific subunit was coexpressed with histone H3 and precipitated complexes were either untreated (-) or treated (+) with ethidium bromide (EtBr) or RNase A or DNase I. Western blot analysis of Nickel affinity purified complexes are shown for (**A**) His_6-Rrn5, (**B**) His_6-Rrn10, (**C**), His_6-Rrn9, and (**D**) His_6-Uaf30-FLAG complexes.

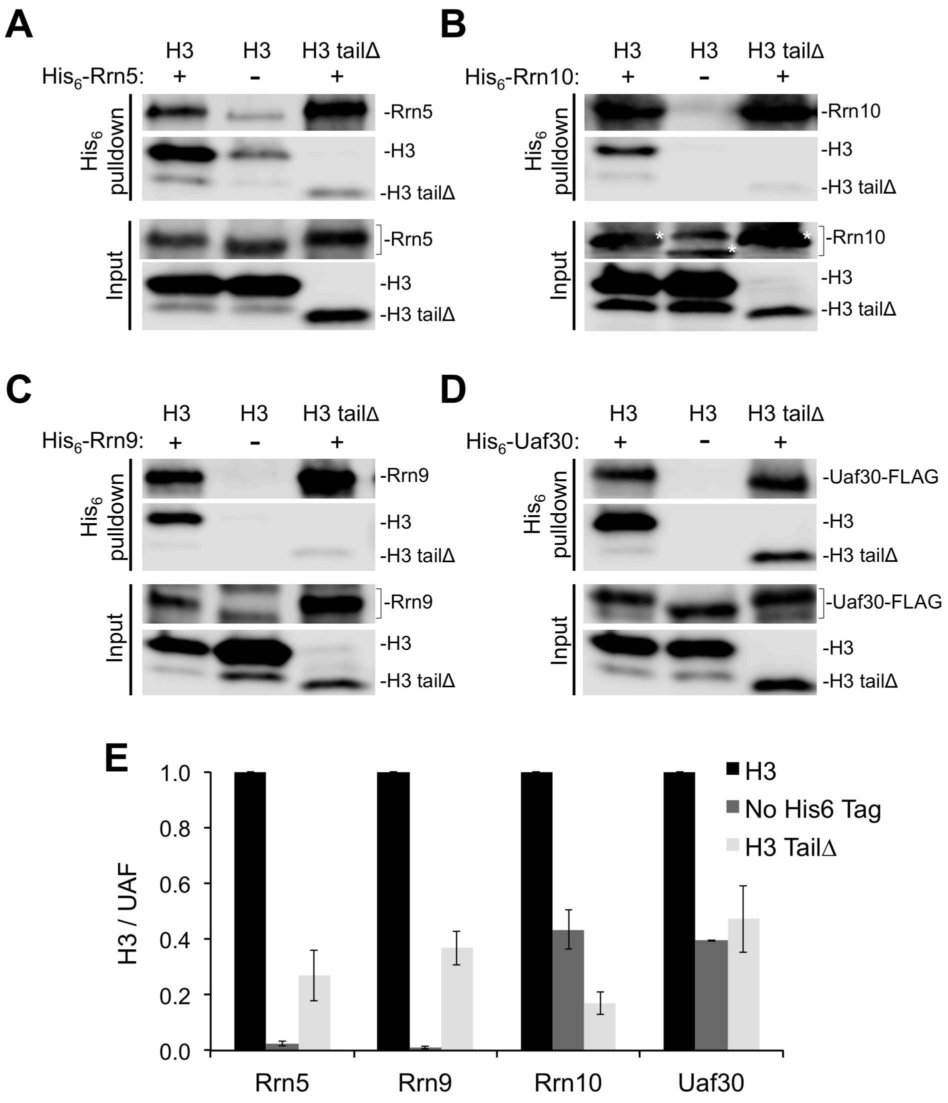

Figure S5. Histone H3 tail domain is necessary for UAF subunit interaction. Each UAF-specific subunit with or without a His_6-tag was coexpressed in *E. coli* with either the full-length H3 protein or H3 lacking its N-terminal tail domain (H3 tailΔ). Western blot analysis of Nickel affinity purified complexes are shown for (**A**) His_6-Rrn5, (**B**) His_6-Rrn10, (**C**) His_6-Rrn9, and (**D**) His_6-Uaf30-FLAG complexes. White asterisks distinguish Rrn10 from a co-migrating background band. (**E**). Relative (Rel.) protein levels of the pulldown assays were plotted in a graph and were normalized against full-length H3, which was set to 1.0. Western blot assays were performed in duplicate and representative assay results are shown. Error bars denote standard deviation.

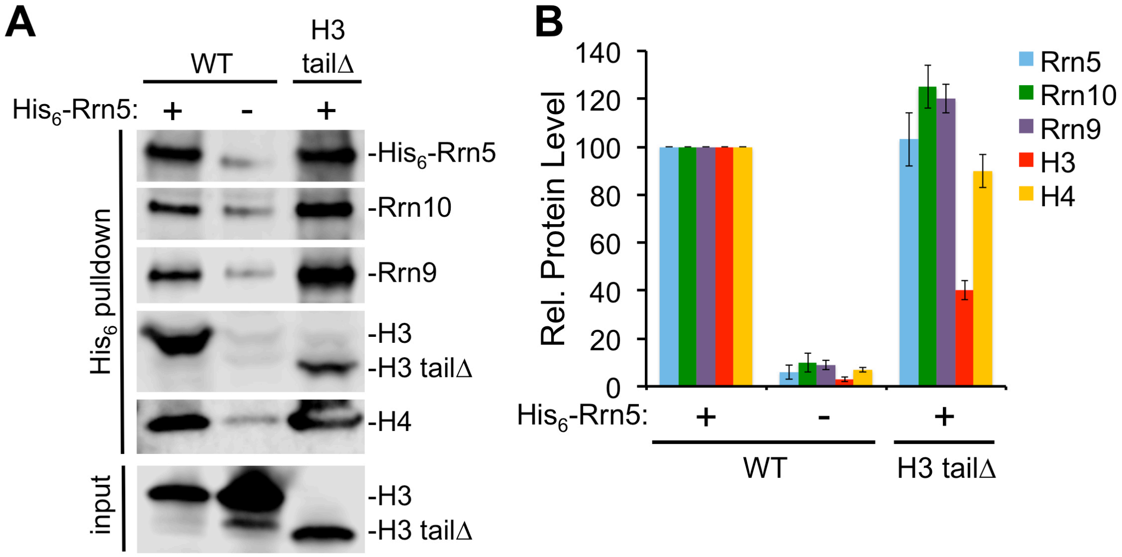

Figure S6. H3 tail domain is necessary for UAF complex association. (**A**). Western blot analysis of isolated UAF complexes pulled down by His_6-tagged Rrn5. UAF was pulled down in the presence of all subunits (WT), with or without a His_6 tag and either full-length or tailΔ histone H3. (**B**). Relative (Rel.) protein levels for each UAF subunit were normalized against WT, which was set at 100. Western assays were performed in duplicate and representative assay results are shown. Error bars denote standard deviation.

Table S1. Plasmids used in this study.

Plasmid	Description
pMS1	pETDuet that expresses His_6-Rrn9, His_6-Uaf30, and His_6-Rrn10
pMS2	pCDFDuet that expresses histone H3, His_6-Rrn5, and histone H4
pMS142	pETDuet that expresses His_6-Rrn9 and His_6-Rrn10
pMS145	pETDuet that expresses Rrn9 and Rrn10
pMS47	pETDuet that expresses Rrn9
pMS154	pCDFDuet that expresses histone H3, histone H4, and Rrn5
pMS170	pETDuet that expresses Rrn10
pMS144	pETDuet that expresses Rrn9 and His_6-Rrn10
pMS28	pETDuet that expresses His_6-Rrn10
pMS51	pCDFDuet that expresses histone H3 and histone H4
pMS143	pETDuet that expresses His_6-Rrn9 and Rrn10
pMS29	pETDuet that expresses His_6-Rrn9
pMS169	pETDuet that expresses Uaf30-Flag
pMS174	pETDuet that expresses His_6-Uaf30-Flag
pMS41	pETDuet that expresses Rrn5
pMS21	pETDuet that expresses His_6-Rrn5
pMS5	pCDFDuet that expresses Histone H3
pMS180	pCDFDuet that expresses His_6-Rrn5
pMS53	pCDFDuet that expresses H3tailΔ (a.a. 29-136)
pMS137	pCDFDuet that expresses His_6-Rrn5 and histone H4
pMS18	pCDFDuet that expresses histone H3 and His_6-Rrn5
pMS206	pACYCDuet that expresses His_6-histone H3
pMS207	pACYCDuet that expresses His_6-histone H4
pMS172	pCDFDuet that expresses H3tailΔ (a.a. 29-136), His_6-Rrn5, and histone H4
pMS207	pCDFDuet that expresses H3tailΔ (a.a. 29-136) and His_6-Rrn5

All plasmids used in this study are available upon request.
